# Association of systemic inflammatory biomarkers with prostate cancer risk: a population-based (NHANES) and clinical validation study

**DOI:** 10.3389/fendo.2025.1697617

**Published:** 2025-11-10

**Authors:** Guoqiang Huang, Kaiwen Xiao, Shuangquan Lin, Xiongbing Lu

**Affiliations:** Department of Urology, The Second Affiliated Hospital of Nanchang University, Nanchang, Jiangxi, China

**Keywords:** prostate cancer, systemic inflammation, NHANES, PSA, NLR, SIRI

## Abstract

**Objective:**

To evaluate the associations between systemic inflammatory biomarkers—systemic immune-inflammation index (SII), systemic inflammation response index (SIRI), pan-immune inflammation value (PIV), neutrophil-to-lymphocyte ratio (NLR), lymphocyte-to-monocyte ratio (LMR), and platelet-to-lymphocyte ratio (PLR)—and prostate cancer (PCa) risk, and to assess their potential for risk in both general and clinical populations.

**Methods:**

A dual-cohort study was conducted using data from the National Health and Nutrition Examination Survey (NHANES; 2001–2010; N=7,354 males, 514 were classified as PCa) and a clinical validation cohort from the second affiliated hospital of Nanchang University (N=353, 175 with biopsy-confirmed PCa). Multivariable logistic regression, restricted cubic spline (RCS) analysis, and receiver operating characteristic (ROC) curve analysis were employed to examine linear/nonlinear relationships and predictive performance of the biomarkers. Models were adjusted for demographic, clinical, and laboratory covariates.

**Results:**

Elevated SII, NLR, PLR, SIRI, and PIV were significantly associated with increased PCa risk in both cohorts, while higher LMR was protective. In the clinical cohort, the highest quartile of SIRI (OR=6.265, 95% CI: 3.130–13.012) and PIV (OR=6.638, 95% CI: 3.343–13.665) showed the strongest risks. RCS analyses revealed nonlinear relationships between biomarkers and PCa risk, total PSA (tPSA), and free PSA (fPSA). Elevated SII, NLR, PLR, SIRI, and PIV were significantly associated with increased PCa risk in both cohorts, while a higher LMR was protective. In the clinical cohort, the highest quartile of SIRI (OR=6.265, 95% CI: 3.130–13.012) and PIV (OR=6.638, 95% CI: 3.343–13.665) exhibited the strongest risks. RCS analyses revealed nonlinear relationships between biomarkers and PCa risk, total PSA (tPSA), and free PSA (fPSA). ROC analysis indicated moderate discriminatory power for PIV (AUC=0.709, 95% CI: 0.655–0.763) and SIRI (AUC=0.704, 95% CI: 0.650–0.759) compared with tPSA in the clinical cohort. However, fPSA and SIRI did not demonstrate a clear advantage, and the DeLong test showed no significant statistical difference.

**Conclusion:**

Systemic inflammatory biomarkers, particularly composite indices such as SIRI and PIV, are strongly associated with PCa risk and demonstrate nonlinear relationships with PSA parameters. These biomarkers may enhance risk stratification for PCa and serve as non-invasive tools to complement existing diagnostic approaches.

## Introduction

Prostate cancer (PCa) is a leading cause of cancer-related mortality among men worldwide, and early detection is critical for improving outcomes, particularly in high-risk cases (1, 2). Current screening relies heavily on prostate-specific antigen (PSA) testing, but its limited specificity, especially in the PSA “gray zone“ (4–20 ng/mL), often results in unnecessary biopsies or missed diagnoses (3, 4). Chronic inflammation, a hallmark of cancer, orchestrates a pro-tumorigenic microenvironment through immune cell dysregulation and cytokine signaling, driving PCa initiation and progression (5–7). Systemic inflammatory biomarkers, such as the systemic immune-inflammation index (SII), lymphocyte-to-monocyte ratio (LMR), systemic inflammation response index (SIRI), neutrophil-to-lymphocyte ratio (NLR), pan-immune inflammation value (PIV), and platelet-to-lymphocyte ratio (PLR), serve as accessible proxies for immune dynamics and have emerged as promising tools for cancer risk assessment and prognosis. For instance, studies have identified a correlation between the expression levels of inflammatory markers, such as SII, SIRI, and NLR, in rectal cancer patients and the subsequent development and progression of the disease (8, 9). Further research highlights the predictive utility of these indices across various solid tumors, suggesting a generalized role of systemic inflammation in oncogenesis.

Evidence suggests that elevated systemic inflammation, as measured by these biomarkers, is associated with increased PCa risk. For instance, a study using the National Health and Nutrition Examination Survey (NHANES) reported a 168% increased PCa risk with elevated SII (odds ratio [OR] = 2.68, 95% confidence interval [CI]: 1.32-5.46) in U.S. men (10). While these biomarkers have shown prognostic value in malignancies such as colorectal cancer, their role in PCa, particularly high-risk cases, remains underexplored. Furthermore, the nature of their associations with PSA parameters (total PSA [tPSA] and free PSA [fPSA])—whether linear or nonlinear—and their clinical utility in PCa screening and risk stratification are not well-established.

This study employs a dual-cohort design, integrating population-based data from NHANES with retrospective clinical data from a tertiary hospital in China, to investigate the associations of six systemic inflammatory biomarkers (SII, SIRI, PIV, NLR, LMR, and PLR) with PCa risk. Using advanced statistical approaches, including receiver operating characteristic (ROC) curve analysis and restricted cubic spline (RCS) regression, we aim to elucidate linear and nonlinear relationships between these biomarkers and PCa risk, as well as their predictive potential. By bridging knowledge gaps in the role of systemic inflammation in PCa, this study seeks to inform early detection strategies and personalized immunotherapeutic approaches, aligning with the growing emphasis on immune-based interventions in cancer management.

## Methods

### Study populations

#### NHANES cohort

**Figure 1:**
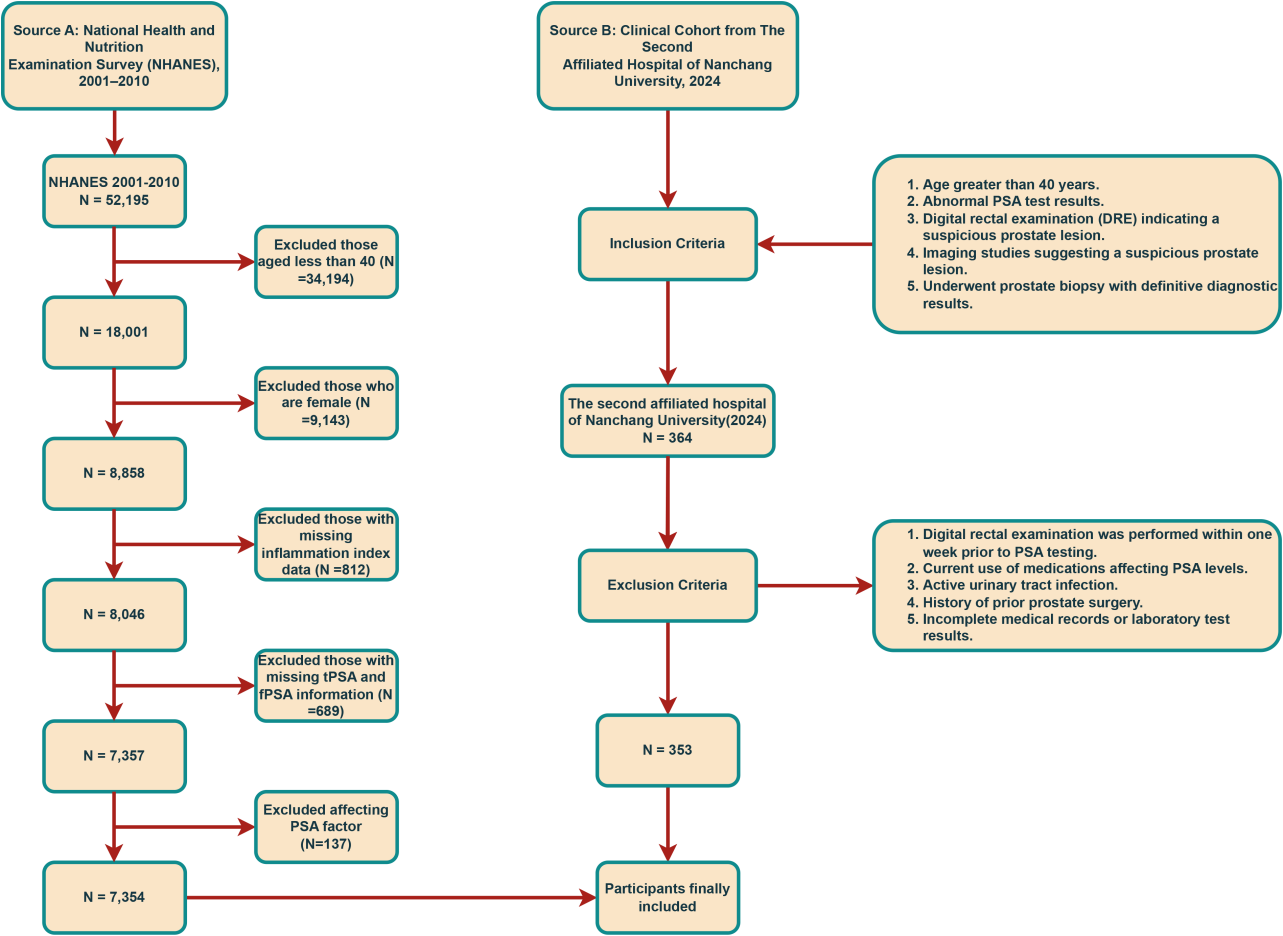
Flow chart for the selection of included sample.

Data for this study were sourced from the NHANES, a comprehensive survey evaluating the health and nutritional status of individuals across the United States. NHANES ensures data quality through self-reported diagnoses, medical record validation, embedded validation questions, and regular quality control checks. We included 52,195 participants from the 2001–2010 cycles, excluding those with incomplete data (N=45,841), resulting in 7,354 male participants (514 were classified as PCa, 6,840 without). The participant selection process is detailed in **Figure 1**. The study protocol was approved by the NCHS Ethics Review Board, and written informed consent was secured from all participants. No additional external ethical approval was required. Data and study details are available at https://www.cdc.gov/nchs/nhanes.

#### Clinical cohort

To validate findings, we analyzed retrospective data from 353 patients undergoing prostate biopsy at the East Lake Campus of the Second Affiliated Hospital of Nanchang University in 2024 (175 with PCa, 178 without). Patients were classified as PCa or Non-PCa based on biopsy-confirmed pathology (prostate acinar adenocarcinoma or variants). Ethics approval for the study was granted by the Ethics Committee of the principal investigator’s institution, The Second Affiliated Hospital of Nanchang University (IIT-O-2025-275). Written informed consent was obtained from all participants at admission.

### Inflammatory biomarker definitions

Inflammatory indices were calculated from peripheral blood data. For the NHANES cohort, complete blood counts provided neutrophil, monocyte, lymphocyte, and platelet counts. For the clinical cohort, indices were derived from routine blood tests at initial consultation. The six biomarkers were calculated as follows:

PIV: (Monocyte count × Platelet count × Neutrophil count) ÷ Lymphocyte count
SII: (Platelet count × Neutrophil count) ÷ Lymphocyte count
NLR: Neutrophil count ÷ Lymphocyte count
LMR: Lymphocyte count ÷ Monocyte count
SIRI: (Monocyte count × Neutrophil count) ÷ Lymphocyte count
PLR: Platelet count ÷ Lymphocyte count

### Prostate cancer assessment

In NHANES, high-risk PCa was defined as tPSA >10 ng/mL or tPSA 4–10 ng/mL with free-to-total PSA ratio (f/t PSA) ≤25%, based on established risk stratification criteria (11). In the clinical cohort, PCa was confirmed by biopsy pathology (acinar adenocarcinoma or variants).

### Covariates

NHANES covariates included biochemical markers (e.g., alanine aminotransferase, triglycerides), poverty income ratio (PIR), age, alcohol use, body mass index (BMI), race/ethnicity, education, blood pressure, smoking status, diabetes history and marital status. Clinical cohort covariates included age, BMI, Types of health insurance, white blood cell count, urea, creatinine, total calcium, estimated glomerular filtration rate (eGFR), potassium, uric acid, and glucose, obtained from routine blood and biochemical tests at initial consultation.

### Statistical analysis

This study analyzed data from both a clinical cohort and NHANES database. The clinical cohort was derived from electronic medical records, with participants excluded if any data were missing. For the NHANES dataset, missing values were imputed using the “mice” R package, and sensitivity analyses confirmed no significant differences between imputed and complete-case datasets (*P* > 0.05; see **Supplementary Materials**). To assess baseline characteristics across different groups, categorical variables were analyzed with chi - square tests, and continuous variables were examined using T - tests. The Shapiro - Wilk test was employed to evaluate the normality of continuous variables. For normally distributed continuous variables, data were presented as the mean ± standard deviation (SD); for non - normally distributed ones, the median (interquartile range [IQR]) was reported. Categorical variables were described as counts (along with their corresponding percentages). Inflammatory indices were partitioned into quartiles: Q1 (values below the 25th percentile), Q2 (ranging from the 25th to 50th percentile), Q3 (from the 50th to 75th percentile), and Q4 (above the 75th percentile). Univariate logistic regression was used to explore the crude associations between inflammatory indices and PCa. Three models were developed for multivariable logistic regression: Model 1 (unadjusted), Model 2 (adjusted for age, race, and marital status), and Model 3 (adjusted for all covariates, including clinical and laboratory parameters). Due to data availability constraints in the retrospective clinical cohort, adjustments were limited to age, BMI, and select laboratory parameters, which may introduce residual confounding. RCS regression with four knots was utilized to investigate the dose - response relationships between inflammatory indices and PCa, tPSA, and fPSA. ROC curve analysis combined with DeLong’s test was used to assess the discriminatory ability of each index for PCa risk. All statistical analyses were carried out using R software (version 4.5.1), and a two - sided *P* - value less than 0.05 was regarded as statistically significant.

## Results

### Baseline characteristics

This dual-cohort study included 7,707 participants, comprising a population-based cohort from NHANES (N=7,354) and an independent clinical cohort (N=353) to validate findings and enhance generalizability.

In the NHANES cohort, 514 participants (7.0%) were classified as PCa. **Table 1** summarizes baseline characteristics, revealing significant differences between Non-PCa and PCa groups. Participants with PCa were older (median age: 71.0 vs. 57.0 years, *P*<0.001). Five inflammatory indices were significantly increased in the PCa group compared to controls (all *P*<0.001): NLR (2.44 vs. 2.07), SII (549 vs. 484), PLR (136 vs. 122), SIRI (1.33 vs. 1.13), and PIV (299 vs. 265). Conversely, LMR was lower in the PCa group (3.12 vs. 3.50, *P*< 0.001), indicating a heightened inflammatory state. tPSA and fPSA levels were significantly higher in the PCa group (tPSA: 6.72 vs. 0.91 ng/mL; fPSA: 1.07 vs. 0.27 ng/mL, *P*<0.001).

**Table 1:** Baseline characteristics of study participants based on the presence of prostate cancer from NHANES 2001-2010.

Characteristic	[ALL]	None-PCa	PCa	P. overall
	N=7354	N=6840	N=514	
Age	59.0 [49.0;70.0]	57.0 [48.0;69.0]	71.0 [62.2;78.0]	<0.001
Race:				0.011
Mexican American	1294 (17.6%)	1226 (17.9%)	68 (13.2%)	
Other Hispanic	441 (6.00%)	410 (5.99%)	31 (6.03%)	
Non-Hispanic White	4025 (54.7%)	3742 (54.7%)	283 (55.1%)	
Non-Hispanic Black	1334 (18.1%)	1217 (17.8%)	117 (22.8%)	
Other Race	260 (3.54%)	245 (3.58%)	15 (2.92%)	
Education:				0.031
Below high school	2310 (31.4%)	2122 (31.0%)	188 (36.6%)	
High school or comparable	1702 (23.1%)	1595 (23.3%)	107 (20.8%)	
College or above	3342 (45.4%)	3123 (45.7%)	219 (42.6%)	
Marital:				0.002
Married/Living with partner	5359 (72.9%)	5015 (73.3%)	344 (66.9%)	
Widowed/Divorced/Separated/	1995 (27.1%)	1825 (26.7%)	170 (33.1%)	
PIR	2.54 [1.29;4.62]	2.55 [1.29;4.62]	2.36 [1.24;4.37]	0.185
BMI	28.0 [25.1;31.4]	28.1 [25.2;31.5]	27.0 [24.0;30.1]	<0.001
Alcohol:				0.023
No	1283 (17.4%)	1174 (17.2%)	109 (21.2%)	
Yes	6071 (82.6%)	5666 (82.8%)	405 (78.8%)	
Diabetes:				0.172
No	6029 (82.0%)	5592 (81.8%)	437 (85.0%)	
Borderline	175 (2.38%)	164 (2.40%)	11 (2.14%)	
Yes	1150 (15.6%)	1084 (15.8%)	66 (12.8%)	
SBP	127 [116;139]	126 [116;139]	132 [118;146]	<0.001
DBP	73.0 [65.0;80.0]	73.0 [65.0;80.0]	72.5 [62.2;79.8]	0.007
NLR	2.09 [1.56;2.81]	2.07 [1.55;2.78]	2.44 [1.75;3.27]	<0.001
SII	487 [346;685]	484 [343;678]	549 [390;789]	<0.001
PLR	122 [96.2;158]	122 [95.9;156]	136 [104;176]	<0.001
LMR	3.50 [2.67;4.50]	3.50 [2.74;4.50]	3.12 [2.29;4.00]	<0.001
SIRI	1.14 [0.78;1.67]	1.13 [0.78;1.64]	1.33 [0.85;2.05]	<0.001
PIV	267 [174;409]	265 [173;404]	299 [190;484]	<0.001
Physical:				0.003
Inactive	3535 (48.1%)	3255 (47.6%)	280 (54.5%)	
Moderate	2284 (31.1%)	2127 (31.1%)	157 (30.5%)	
Vigorous	615 (8.36%)	584 (8.54%)	31 (6.03%)	
Both moderate and vigorous	920 (12.5%)	874 (12.8%)	46 (8.95%)	
tPSA	1.00 [0.60;1.90]	0.91 [0.56;1.61]	6.72 [4.92;10.4]	<0.001
fPSA	0.29 [0.18;0.49]	0.27 [0.17;0.43]	1.07 [0.78;1.65]	<0.001
ALT	24.0 [19.0;32.0]	24.0 [19.0;32.0]	21.0 [17.0;26.0]	<0.001
AST	25.0 [21.0;30.0]	25.0 [21.0;30.0]	24.0 [20.0;28.0]	<0.001
TG	1.52 [1.02;2.34]	1.53 [1.03;2.35]	1.35 [0.95;2.03]	<0.001
Smoke:				0.009
Never	2760 (37.5%)	2562 (37.5%)	198 (38.5%)	
Former	2902 (39.5%)	2677 (39.1%)	225 (43.8%)	
Current	1692 (23.0%)	1601 (23.4%)	91 (17.7%)	

PCa, Prostate cancer; PIR, Poverty income ratio; BMI, body mass index; WBC, white blood cell count; tPSA, Total prostatic specific antigen; fPSA, free prostatic specific antigen; ALT, Alanine aminotransferase; AST, Aspartate aminotransferase; TG, Triglycerides; SBP, Systolic blood pressure; DBP, Diastolic blood pressure.

The clinical validation cohort included 353 participants, with 175 (49.6%) classified as having PCa. The groups were balanced for BMI and insurance status (*P* > 0.05). Consistent with NHANES findings, all inflammatory indices were significantly dysregulated in the positive outcome group (**Table 2**). This group exhibited higher neutrophil counts (5.33 vs. 4.30 ×10³/µL, *P*<0.001), monocyte counts (0.57 vs. 0.45 ×10³/µL, *P*<0.001), and platelet counts (224 vs. 194 ×10³/µL, *P*=0.001), resulting in elevated SII (865 vs. 628), NLR (3.90 vs. 3.04), PLR (155 vs. 139), SIRI (2.22 vs. 1.25), and PIV (503 vs. 272) (all *P* ≤ 0.001). LMR was lower (2.33 vs. 3.30, *P*<0.001), and PSA levels were substantially higher (tPSA: 38.1 vs. 11.0 ng/mL; fPSA: 6.21 vs. 1.69 ng/mL, *P*<0.001).

**Table 2:** Baseline characteristics of study participants based on the presence of prostate cancer from clinical cohort.

Characteristic	[ALL]	None-PCa	PCa	P. overall
	N=353	N=178	N=175	
BMI	23.6 [21.2;25.8]	23.9 [21.5;26.2]	23.4 [20.7;25.2]	0.067
Healthcare:				0.482
Private insurance	208 (58.9%)	108 (60.7%)	100 (57.1%)	
Public insurance	144 (40.8%)	69 (38.8%)	75 (42.9%)	
Without insurance	1 (0.28%)	1 (0.56%)	0 (0.00%)	
Age	72.0 [67.0;77.0]	71.0 [66.0;76.0]	73.0 [68.5;78.0]	0.004
Neutrophil	4.88 [3.68;6.40]	4.30 [3.39;5.77]	5.33 [4.36;6.99]	<0.001
Monocyte	0.51 [0.39;0.67]	0.45 [0.34;0.60]	0.57 [0.47;0.72]	<0.001
Lymphocyte	1.39 [1.09;1.79]	1.44 [1.15;1.84]	1.33 [1.02;1.71]	0.107
WBC	6.56 [5.36;8.42]	6.54 [5.31;8.40]	6.61 [5.39;8.46]	0.763
PLT	209 [175;256]	194 [170;244]	224 [184;270]	0.001
AST	23.4 [19.8;28.8]	23.0 [19.9;29.0]	23.8 [19.6;28.7]	0.939
ALT	19.0 [14.0;27.0]	21.0 [14.9;29.0]	17.9 [13.9;26.0]	0.020
Urea	5.91 [4.86;7.53]	5.44 [4.29;7.00]	6.00 [5.00;8.12]	0.001
Creatinine	85.5 [75.0;99.3]	85.5 [75.9;98.9]	85.0 [73.3;100]	0.735
eGFR	82.0 [70.2;92.8]	82.0 [70.9;92.0]	81.7 [67.3;95.1]	0.945
Uric Acid	365 [311;428]	353 [305;425]	371 [317;434]	0.191
Potassium	4.00 [3.79;4.00]	4.00 [3.81;4.00]	4.00 [3.78;4.00]	0.850
Total Calcium	2.34 [2.25;2.43]	2.34 [2.25;2.43]	2.34 [2.25;2.42]	0.610
Glucose	6.00 [5.02;7.66]	5.93 [5.00;7.68]	6.32 [5.31;7.58]	0.089
tPSA	17.0 [8.91;43.2]	11.0 [6.25;20.4]	38.1 [13.2;100]	<0.001
fPSA	2.73 [1.00;8.93]	1.69 [1.00;3.26]	6.21 [2.00;22.4]	<0.001
SII	734 [484;1164]	628 [414;928]	865 [609;1397]	<0.001
NLR	3.53 [2.43;4.99]	3.04 [2.25;4.64]	3.90 [2.90;6.15]	<0.001
PLR	147 [114;204]	139 [107;184]	155 [126;230]	0.001
LMR	2.78 [1.97;3.74]	3.30 [2.41;4.39]	2.33 [1.70;3.10]	<0.001
SIRI	1.71 [1.08;3.04]	1.25 [0.82;2.41]	2.22 [1.53;3.97]	<0.001
PIV	376 [215;661]	272 [152;514]	503 [327;907]	<0.001

PCa, Prostate cancer; BMI, body mass index; WBC, white blood cell count; tPSA, Total prostatic specific antigen; fPSA, free prostatic specific antigen; ALT, Alanine aminotransferase; AST, Aspartate aminotransferase; eGFR, estimated Glomerular Filtration Rate; PLT, Platelet count.

### Associations of inflammatory indices with PCa risk

As demonstrated in **Table 3**, multivariable logistic regression (Model 3) revealed statistically significant associations between systemic inflammatory indices and PCa risk when evaluated as continuous variables within the NHANES cohort. Higher NLR (OR=1.114, 95% CI: 1.053–1.176, *P*<0.001), SIRI (OR=1.104, 95% CI: 1.027–1.183, *P*=0.006), and PLR (OR=1.004, 95% CI: 1.002–1.005, *P*<0.001) were associated with increased PCa risk. Quartile analysis (Q4 vs. Q1) revealed stronger associations: SII (OR=1.865, 95% CI: 1.417–2.469, *P*<0.001), NLR (OR=1.540, 95% CI: 1.166–2.044, *P*=0.003), PLR (OR=1.665, 95% CI: 1.275–2.184, *P*<0.001), and PIV (OR=1.422, 95% CI: 1.085–1.871, *P*=0.011) significantly increased PCa risk. The association for SIRI was attenuated in Q4 (*P*=0.140). Higher LMR was protective, with Q3 showing reduced PCa odds (OR=0.658, 95% CI: 0.498–0.866, *P*=0.003).

**Table 3:** Associations of inflammatory indices with prostate cancer.

Characteristic		Model 1			Model 2			Model 3		
*US NHANES*		OR	95% CI	*P-Value*	OR	95% CI	*P-Value*	OR	95% CI	*P-Value*
SII	SII	1.000	1.000, 1.000	0.005	1.000	1.000, 1.000	0.018	1.000	1.000, 1.000	0.024
	Q1	—	—		—	—		—	—	
	Q2	1.351	1.028, 1.781	0.032	1.455	1.097, 1.937	0.010	1.500	1.127, 2.003	0.006
	Q3	1.259	0.954, 1.666	0.100	1.323	0.991, 1.769	0.058	1.343	1.003, 1.802	0.048
	Q4	1.931	1.495, 2.509	<0.001	1.827	1.394, 2.407	<0.001	1.865	1.417, 2.469	<0.001
NLR	NLR	1.187	1.127, 1.250	<0.001	1.109	1.049, 1.169	<0.001	1.114	1.053, 1.176	<0.001
	Q1	—	—		—	—		—	—	
	Q2	0.937	0.699, 1.254	0.7	0.948	0.699, 1.284	0.7	0.973	0.716, 1.322	0.900
	Q3	1.414	1.083, 1.852	0.011	1.261	0.951, 1.679	0.11	1.303	0.979, 1.739	0.071
	Q4	2.040	1.588, 2.637	<0.001	1.494	1.136, 1.974	0.004	1.540	1.166, 2.044	0.003
SIRI	SIRI	1.214	1.137, 1.297	<0.001	1.097	1.021, 1.174	0.009	1.104	1.027, 1.183	0.006
	Q1	—	—		—	—		—	—	
	Q2	0.926	0.702, 1.220	0.6	0.870	0.650, 1.163	0.3	0.893	0.666, 1.196	0.400
	Q3	1.031	0.787, 1.351	0.8	0.844	0.631, 1.129	0.3	0.889	0.662, 1.193	0.400
	Q4	1.758	1.379, 2.250	<0.001	1.162	0.883, 1.536	0.3	1.238	0.936, 1.643	0.140
PLR	PLR	1.005	1.004, 1.006	<0.001	1.004	1.003, 1.005	<0.001	1.004	1.002, 1.005	<0.001
	Q1	—	—		—	—		—	—	
	Q2	1.078	0.812, 1.432	0.6	1.050	0.786, 1.405	0.7	1.025	0.765, 1.374	0.900
	Q3	1.352	1.033, 1.776	0.029	1.419	1.076, 1.877	0.014	1.344	1.014, 1.785	0.040
	Q4	1.946	1.512, 2.519	<0.001	1.754	1.351, 2.288	<0.001	1.665	1.275, 2.184	<0.001
PIV	PIV	1.000	1.000, 1.001	0.016	1.000	1.000, 1.000	0.044	1.000	1.000, 1.000	0.049
	Q1	—	—		—	—		—	—	
	Q2	1.001	0.762, 1.313	>0.9	0.995	0.750, 1.320	>0.9	1.002	0.754, 1.332	>0.9
	Q3	1.058	0.809, 1.385	0.7	1.028	0.775, 1.363	0.9	1.069	0.804, 1.422	0.600
	Q4	1.637	1.281, 2.101	<0.001	1.365	1.046, 1.787	0.023	1.422	1.085, 1.871	0.011
LMR	LMR	0.849	0.793, 0.906	<0.001	0.970	0.913, 1.026	0.3	0.970	0.912, 1.026	0.300
	Q1	—	—		—	—		—	—	
	Q2	0.619	0.491, 0.778	<0.001	0.825	0.648, 1.047	0.12	0.811	0.636, 1.032	0.090
	Q3	0.408	0.314, 0.526	<0.001	0.657	0.498, 0.861	0.003	0.658	0.498, 0.866	0.003
	Q4	0.448	0.345, 0.577	<0.001	0.771	0.578, 1.021	0.072	0.763	0.570, 1.016	0.066
Clinical cohort										
SII	SII	1.001	1.000, 1.001	<0.001	1.000	1.000, 1.001	0.003	1.000	1.000, 1.001	0.002
	Q1	—	—		—	—		—	—	
	Q2	2.446	1.321, 4.607	0.005	2.063	1.091, 3.952	0.027	2.171	1.128, 4.241	0.021
	Q3	3.216	1.737, 6.079	<0.001	2.752	1.459, 5.285	0.002	2.684	1.406, 5.214	0.003
	Q4	4.949	2.644, 9.512	<0.001	4.057	2.113, 7.969	<0.001	4.119	2.119, 8.198	<0.001
NLR	NLR	1.129	1.048, 1.225	0.002	1.100	1.020, 1.194	0.016	1.109	1.026, 1.205	0.012
	Q1	—	—		—	—		—	—	
	Q2	2.423	1.313, 4.543	0.005	2.154	1.152, 4.084	0.017	2.013	1.060, 3.868	0.034
	Q3	3.188	1.726, 6.004	<0.001	2.824	1.508, 5.380	0.001	2.773	1.458, 5.369	0.002
	Q4	4.038	2.176, 7.668	<0.001	3.322	1.744, 6.450	<0.001	3.319	1.719, 6.530	<0.001
SIRI	SIRI	1.143	1.053, 1.261	0.004	1.115	1.032, 1.225	0.012	1.124	1.037, 1.238	0.009
	Q1	—	—		—	—		—	—	
	Q2	3.944	2.057, 7.814	<0.001	3.499	1.806, 6.987	<0.001	3.296	1.672, 6.690	<0.001
	Q3	6.903	3.574, 13.856	<0.001	6.214	3.182, 12.592	<0.001	6.052	3.057, 12.436	<0.001
	Q4	7.253	3.749, 14.591	<0.001	6.033	3.056, 12.340	<0.001	6.265	3.130, 13.012	<0.001
PLR	PLR	1.004	1.002, 1.007	0.002	1.003	1.001, 1.006	0.024	1.003	1.001, 1.006	0.019
	Q1	—	—		—	—		—	—	
	Q2	1.351	0.741, 2.474	0.3	1.146	0.616, 2.137	0.7	1.041	0.549, 1.972	>0.9
	Q3	2.036	1.121, 3.738	0.020	1.655	0.888, 3.108	0.11	1.584	0.836, 3.018	0.2
	Q4	2.828	1.548, 5.249	<0.001	2.283	1.209, 4.362	0.011	2.273	1.184, 4.418	0.014
PIV	PIV	1.001	1.000, 1.001	<0.001	1.001	1.000, 1.001	<0.001	1.001	1.000, 1.001	<0.001
	Q1	—	—		—	—		—	—	
	Q2	3.364	1.764, 6.599	<0.001	2.971	1.541, 5.878	0.001	2.699	1.378, 5.418	0.004
	Q3	5.851	3.058, 11.591	<0.001	5.159	2.666, 10.314	<0.001	4.877	2.477, 9.916	<0.001
	Q4	7.895	4.082, 15.869	<0.001	6.711	3.416, 13.661	<0.001	6.638	3.343, 13.665	<0.001
LMR	LMR	0.545	0.447, 0.656	<0.001	0.571	0.465, 0.692	<0.001	0.558	0.450, 0.680	<0.001
	Q1	—	—		—	—		—	—	
	Q2	0.569	0.303, 1.056	0.076	0.609	0.322, 1.142	0.12	0.552	0.283, 1.062	0.077
	Q3	0.413	0.220, 0.762	0.005	0.443	0.234, 0.828	0.011	0.399	0.205, 0.764	0.006
	Q4	0.099	0.048, 0.195	<0.001	0.117	0.055, 0.237	<0.001	0.104	0.048, 0.217	<0.001

NHANES Cohort:

Model 1: Unadjusted baseline model. Model 2: Adjusted for Age, Race, and Marital Status. Model 3: Adjusted for Age, Race, Marital Status, Education, Poverty Income Ratio (PIR), Body Mass Index (BMI), Smoking status, Alcohol consumption, Physical activity, Diabetes status, Systolic Blood Pressure (SBP), Diastolic Blood Pressure (DBP), Triglycerides (TG), Alanine Aminotransferase (ALT), and Aspartate Aminotransferase (AST).

Clinical Cohort: Model 1: Unadjusted baseline model. Model 2: Adjusted for Age, BMI, and healthcare access/utilization. Model 3: Adjusted for Age, BMI, healthcare access/utilization, White Blood Cell count (WBC), AST, ALT, Urea, Creatinine, estimated Glomerular Filtration Rate (eGFR), Uric Acid, Potassium, Total Calcium, and Glucose.

OR, odds ratio; CI, confidence interval; PCa, Prostate cancer; PIR, Poverty income ratio; BMI, body mass index; WBC, white blood cell count; tPSA, Total prostatic specific antigen; fPSA, free prostatic specific antigen; ALT, Alanine aminotransferase; AST, Aspartate aminotransferase; TG, Triglycerides; SBP, Systolic blood pressure; DBP, Diastolic blood pressure; eGFR, estimated Glomerular Filtration Rate; PLT, Platelet count.

In the clinical cohort, associations were stronger and more consistent. All inflammatory indices, analyzed continuously in Model 3, were significantly associated with PCa: SII (OR=1.000, 95% CI: 1.000–1.001, *P*=0.002), NLR (OR=1.109, 95% CI: 1.026–1.205, *P*=0.012), SIRI (OR=1.124, 95% CI: 1.037–1.238, *P*=0.009), PLR (OR=1.003, 95% CI: 1.001–1.006, *P*=0.019), PIV (OR=1.001, 95% CI: 1.000–1.001, *P*<0.001), and LMR (OR=0.558, 95% CI: 0.450–0.680, *P*<0.001). Quartile analysis demonstrated a dose-response relationship, with Q4 showing notably elevated risks: SII (OR=4.119, 95% CI: 2.119–8.198, *P*<0.001), NLR (OR=3.319, 95% CI: 1.719–6.530, *P*<0.001), SIRI (OR=6.265, 95% CI: 3.130–13.012, *P*<0.001), PIV (OR=6.638, 95% CI: 3.343–13.665, *P*<0.001), and PLR (OR=2.273, 95% CI: 1.184–4.418, *P*=0.014). Higher LMR was strongly protective (Q4 vs. Q1: OR=0.104, 95% CI: 0.048–0.217, *P*<0.001).

### Nonlinear associations with PCa risk

**Figure 2:**
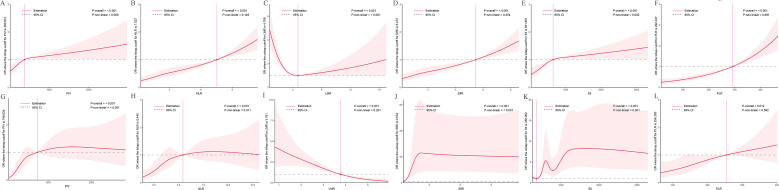
Dose-response relationships between inflammatory indices and PCa risk. The solid red line represents the estimated odds ratio (OR), and the shaded area indicates the 95% confidence interval (CI). The dashed horizontal gray line marks an OR of 1, indicating no association. The vertical pink line highlights a specific cutoff value or inflection point for each inflammatory index, as denoted in the Y-axis label. Statistical significance for the overall association and non-linearity is provided (P-overall and P-non-linear, respectively). **(A-F)** Data derived from the NHANES cohort. **(G-L)** Data derived from the independent clinical validation cohort. **(A)** PIV in NHANES; **(B)** NLR in NHANES; **(C)** LMR in NHANES; **(D)** SIRI in NHANES; **(E)** SII in NHANES; **(F)** PLR in NHANES; **(G)** PIV in clinical cohort; **(H)** NLR in clinical cohort; **(I)** LMR in clinical cohort; **(J)** SIRI in clinical cohort; **(K)** SII in clinical cohort; **(L)** PLR in clinical cohort.

RCS analyses explored nonlinear associations between inflammatory indices and PCa risk (**Figure 2**). In the NHANES cohort (**Figures 2A–F**), PIV, NLR, SIRI, SII, and PLR showed positive, dose-dependent associations with PCa risk (all *P*-overall < 0.001). Nonlinearity was evident for PIV (*P*-nonlinear = 0.008) and SII (*P*-nonlinear = 0.002), while NLR (*P*-nonlinear = 0.149), SIRI (*P*-nonlinear = 0.254), and PLR (*P*-nonlinear = 0.981) were largely linear. LMR exhibited a U-shaped relationship (*P*-overall < 0.001, *P*-nonlinear < 0.001), with both low and high values associated with increased PCa risk and an optimal range linked to lower risk.

In the clinical cohort (**Figures 2G–L**), similar patterns were observed, reinforcing NHANES findings. PIV (*P*-overall < 0.001, *P*-nonlinear < 0.001), NLR (*P*-overall < 0.001, *P*-nonlinear = 0.011), SIRI (*P*-overall < 0.001, *P*-nonlinear < 0.001), and SII (*P*-overall < 0.001, *P*-nonlinear = 0.001) showed increasing trends with PCa risk. PLR exhibited a weaker but significant association (*P*-overall = 0.012, *P*-nonlinear = 0.592). LMR displayed a negative, nonlinear relationship (*P*-overall < 0.001, *P*-nonlinear = 0.251), with lower values linked to higher risk, though the U-shape was less pronounced than in NHANES.

### Nonlinear associations with tPSA and fPSA

**Figure 3:**
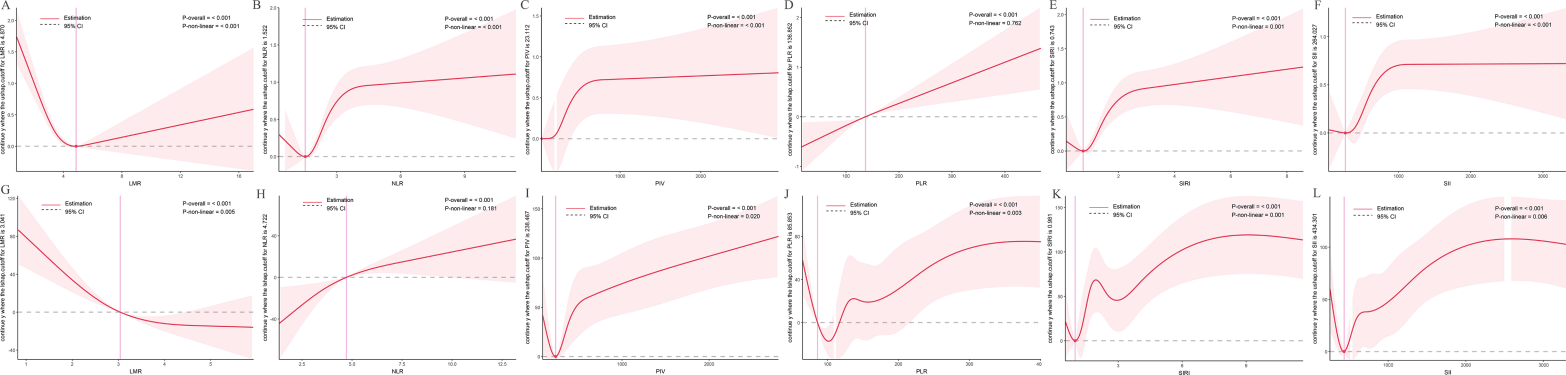
Dose-response relationships between inflammatory indices and total prostate-specific antigen (tPSA) levels. The solid red line represents the estimated spline function value, reflecting the continuous association with tPSA levels. The shaded area indicates the 95% confidence interval (CI). The dashed horizontal gray line at Y=0 indicates the reference point, meaning no estimated change in tPSA levels. The vertical pink line highlights a specific cutoff value or inflection point for each inflammatory index, with its corresponding estimated Y value denoted in the Y-axis label. Statistical significance for the overall association and non-linearity is provided (P-overall and P-non-linear, respectively). **(A-F)** Data derived from the NHANES cohort. **(G-L)** Data derived from the independent clinical validation cohort. **(A)** LMR in NHANES; **(B)** NLR in NHANES; **(C)** PIV in NHANES; **(D)** PLR in NHANES; **(E)** SIRI in NHANES; **(F)** SII in NHANES; **(G)** LMR in clinical cohort; **(H)** NLR in clinical cohort; **(I)** PIV in clinical cohort; **(J)** PLR in clinical cohort; **(K)** SIRI in clinical cohort; **(L)** SII in clinical cohort.

RCS analyses assessed relationships between inflammatory indices and tPSA levels (**Figure 3**). In the NHANES cohort (**Figures 3A–F**), LMR (*P*-overall < 0.001, *P*-nonlinear < 0.001), NLR (*P*-overall < 0.001, *P*-nonlinear < 0.001), SIRI (*P*-overall < 0.001, *P*-nonlinear < 0.001), and SII (*P*-overall < 0.001, *P*-nonlinear < 0.001) exhibited U-shaped associations, with minimum tPSA levels at intermediate values (LMR ~4.070, NLR ~1.522, SIRI ~0.743, SII ~264.027). PIV (*P*-overall < 0.001, *P*-nonlinear < 0.001) showed a complex pattern, with tPSA initially decreasing, then rising steeply, and plateauing. PLR (*P*-overall < 0.001, *P*-nonlinear = 0.762) displayed a linear increase with tPSA.

In the clinical cohort (**Figures 3G–L**), LMR (*P*-overall < 0.001, *P*-nonlinear = 0.005) showed an inverse relationship, with lower LMR linked to higher tPSA. NLR (*P*-overall < 0.001, *P*-nonlinear = 0.181) exhibited a U-shaped trend. PIV (*P*-overall < 0.001, *P*-nonlinear = 0.020), PLR (*P*-overall < 0.001, *P*-nonlinear = 0.003), SIRI (*P*-overall < 0.001, *P*-nonlinear = 0.001), and SII (*P*-overall < 0.001, *P*-nonlinear = 0.006) showed complex nonlinear patterns, with intermediate index values linked to lower tPSA and extremes to higher tPSA.

**Figure 4:**
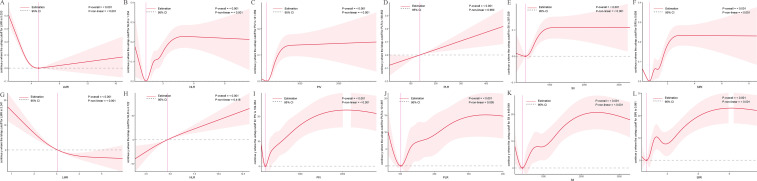
Dose-response relationships between inflammatory indices and free prostate-specific antigen (fPSA) levels. The solid red line represents the estimated spline function value, reflecting the continuous association with fPSA levels. The shaded area indicates the 95% confidence interval (CI). The dashed horizontal gray line at Y=0 indicates the reference point, meaning no estimated change in fPSA levels. The vertical pink line highlights a specific cutoff value or inflection point for each inflammatory index, with its corresponding estimated Y value denoted in the Y-axis label. Statistical significance for the overall association and non-linearity is provided (P-overall and P-non-linear, respectively). **(A-F)** Data derived from the NHANES cohort. **(G-L)** Data derived from the independent clinical validation cohort. **(A)** LMR in NHANES; **(B)** NLR in NHANES; **(C)** PIV in NHANES; **(D)** PLR in NHANES; **(E)** SII in NHANES; **(F)** SIRI in NHANES; **(G)** LMR in clinical cohort; **(H)** NLR in clinical cohort; **(I)** PIV in clinical cohort; **(J)** PLR in clinical cohort; **(K)** SII in clinical cohort; **(L)** SIRI in clinical cohort.

RCS analyses for fPSA (**Figure 4**) revealed similar patterns. In the NHANES cohort (**Figures 4A–F**), LMR (*P*-overall < 0.001, *P*-nonlinear < 0.001), NLR (*P*-overall < 0.001, *P*-nonlinear < 0.001), PIV (*P*-overall < 0.001, *P*-nonlinear < 0.001), SII (*P*-overall < 0.001, *P*-nonlinear < 0.001), and SIRI (*P*-overall < 0.001, *P*-nonlinear < 0.001) showed nonlinear associations, with minimum fPSA at intermediate values (LMR ~5.033, NLR ~1.354, PIV ~141.369, SII ~297.039, SIRI ~0.658). PLR (*P*-overall < 0.001, *P*-nonlinear = 0.980) showed a linear increase.

In the clinical cohort (**Figures 4G–L**), LMR (*P*-overall < 0.001, *P*-nonlinear < 0.001) exhibited an inverse relationship, with lower LMR linked to higher fPSA. NLR (*P*-overall < 0.001, *P*-nonlinear = 0.418) showed a largely linear increase. PIV (*P*-overall < 0.001, *P*-nonlinear < 0.001), PLR (*P*-overall < 0.001, *P*-nonlinear = 0.005), SII (*P*-overall < 0.001, *P*-nonlinear < 0.001), and SIRI (*P*-overall < 0.001, *P*-nonlinear < 0.001) displayed complex nonlinear patterns, with fPSA decreasing initially, then increasing and plateauing at higher values.

### Predictive performance of inflammatory indices

**Figure 5:**
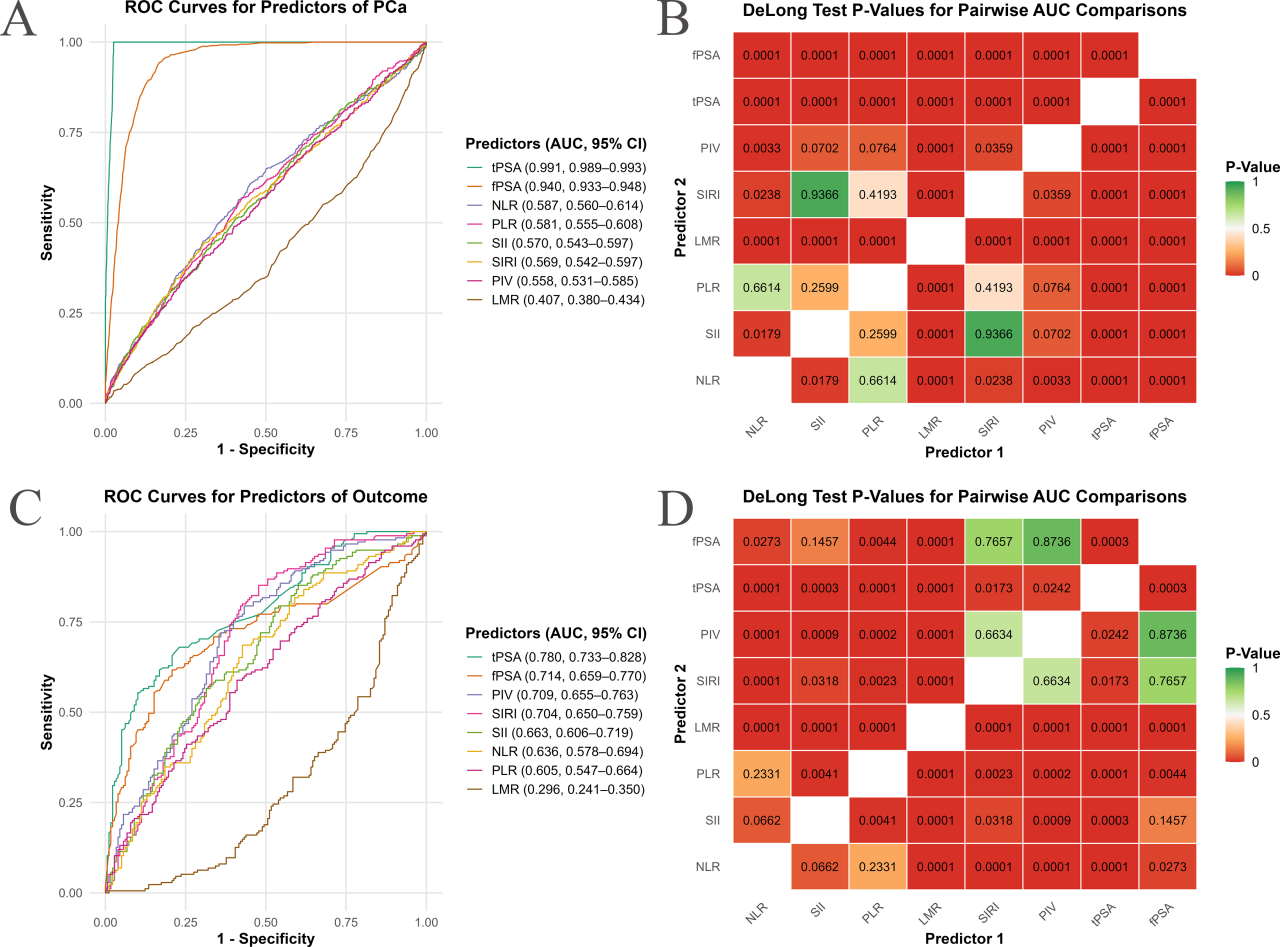
Receiver operating characteristic (ROC) curve analysis of inflammatory indices for PCa risk prediction. This figure assesses the predictive performance of inflammatory indices for PCa risk using ROC curve analysis and Area Under the Curve (AUC) comparisons. **(A, C)** show ROC curves for individual inflammatory indices, plotting sensitivity (true positive rate) against 1-specificity (false positive rate). Each colored line represents an inflammatory index, with its AUC value and 95% confidence interval (CI) listed in the subpanel legend. Curves shifted higher and to the left indicate superior discriminatory ability. **(B, D)** present heatmaps of P-values from DeLong’s test for pairwise AUC comparisons between inflammatory indices. Each cell displays the P-value for the comparison of their AUCs. The color scale ranges from green (P ≥ 0.05, non-significant difference) through orange (intermediate P-values) to red (P < 0.05, significant difference). **(A, B)** represent data from the NHANES cohort, while **(C, D)** are from an independent clinical validation cohort.

ROC curve analysis evaluated the discriminatory ability of inflammatory indices for PCa risk (**Figure 5**). In the NHANES cohort (**Figures 5A, B**), where PCa diagnosis was derived from tPSA and fPSA levels, tPSA [area under the curve (AUC) = 0.991, 95% CI: 0.989–0.993] and fPSA (AUC=0.940, 95% CI: 0.933–0.948) demonstrated high discriminatory power. However, this might introduce statistical bias given the methods of PCa ascertainment. Among the inflammatory indices, AUC values ranged from 0.407 to 0.587, with NLR showing the highest discriminatory ability (AUC=0.587, 95% CI: 0.560–0.614), followed by PLR (0.581, 95% CI: 0.555–0.608), SII (0.570, 95% CI: 0.543–0.597), SIRI (0.569, 95% CI: 0.542–0.597), and PIV (0.558, 95% CI: 0.531–0.585). LMR had the lowest AUC (0.407, 95% CI: 0.380–0.434; *P*<0.001 vs. others, DeLong’s test). Significant differences included NLR vs. SII (*P*=0.0179) and NLR vs. PIV (*P*=0.0033).

In the clinical cohort (**Figures 5C, D**), predictive performance was stronger, tPSA showed an AUC of 0.780 (95% CI: 0.733–0.828), and fPSA had an AUC of 0.714 (95% CI: 0.659–0.770). PIV had the highest AUC (0.709, 95% CI: 0.655–0.763), followed by SIRI (0.704, 95% CI: 0.650–0.759), SII (0.663, 95% CI: 0.606–0.719), NLR (0.636, 95% CI: 0.578–0.694), and PLR (0.605, 95% CI: 0.547–0.664). LMR again showed poor performance (AUC=0.296, 95% CI: 0.241–0.350; *P*<0.001 vs. others). PIV outperformed most indices except SIRI (*P*=0.6634), and SIRI outperformed SII (*P*=0.0318), NLR (*P*=0.0023), and PLR (*P*=0.0001). While tPSA demonstrated a higher AUC, it did not exhibit a distinctly superior advantage when compared to inflammatory indices such as PIV and SIRI. Furthermore, the performance of PIV and fPSA did not show a significant statistical difference (DeLong test P=0.7657).

## Discussion

This dual-cohort study provides evidence linking systemic inflammatory biomarkers (SII, SIRI, PIV, NLR, LMR, and PLR) to PCa risk and PSA parameters (tPSA and fPSA). By integrating population-based data from the NHANES with a biopsy-confirmed clinical cohort from a tertiary hospital in China, we found that elevated pro-inflammatory indices (SII, PIV, SIRI, PLR, NLR) were associated with increased PCa risk, while higher LMR was protective. These associations were more pronounced in the clinical cohort, suggesting a stronger role for systemic inflammation in PCa. Crucially, RCS analyses unveiled intricate nonlinear relationships, offering a nuanced perspective on the dynamic interplay between systemic inflammation and PCa immunobiology, which extends beyond conventional linear assumptions and represents a key novel finding of our study.

In the clinical cohort, SIRI (OR=6.265, 95% CI: 3.130–13.012) and PIV (OR=6.638, 95% CI: 3.343–13.665) in the highest quartile showed the strongest associations with PCa risk, indicating a dose-response effect. Conversely, higher LMR was protective (Q4 vs. Q1: OR=0.104, 95% CI: 0.048–0.217). These findings align with a prior study that reported a 33% increased PCa risk with elevated SIRI (OR=2.57, 95% CI: 1.86–3.54) (12) and suggest that composite indices like SIRI and PIV, which incorporate monocyte counts, may reflect the critical role of monocyte-derived tumor-associated macrophages in PCa progression (13, 14). The protective effect of LMR underscores the importance of lymphocyte-mediated immune surveillance in counteracting pro-tumorigenic inflammation, consistent with the cancer-immunity cycle (6, 15). SIRI and PIV outperform simpler two-cell ratios (e.g., NLR, PLR) likely due to their integration of broader immune cell dynamics, particularly monocytes, which infiltrate the tumor microenvironment (TME) and differentiate into TAMs. These TAMs, often M2-like, promote tumor growth through pro-angiogenic factors, growth factors, and immunosuppressive cytokines, and are enriched in advanced PCa (16, 17). Specifically, M2-like TAMs orchestrate immune evasion by secreting arginase-1 and IL-10, thereby inhibiting T-cell function, and actively participate in extracellular matrix remodeling, facilitating PCa invasion and metastasis (18, 19). The CSF1/CSF1R signaling pathway, a key driver of monocyte recruitment and TAM differentiation, underscores the mechanistic basis for these associations (20, 21).

Systemic inflammation shapes the PCa tumor microenvironment through immune cell interactions and signaling pathways (22–26). Neutrophils, which contribute to NLR, SII, SIRI, and PIV, promote tumor progression via neutrophil extracellular traps and pro-angiogenic factors (27). In contrast, platelets, which contribute to PLR and SII, support tumor cell survival and metastasis (28, 29). Monocytes, reflected in SIRI, PIV, and LMR, differentiate into TAMs that secrete pro-inflammatory cytokines (e.g., IL-6, TNF-α) and immunosuppressive signals (e.g., PD-L1) (30, 31). The NF-κB pathway, activated by inflammatory cascades, drives expression of these cytokines and chemokines, sustaining a pro-tumorigenic TME and promoting epithelial-mesenchymal transition in PCa (32). Beyond its role in TME regulation, persistent activation of NF-κB in PCa cells themselves promotes their survival, proliferation, and resistance to apoptosis, forming a critical feedback loop with inflammatory mediators (33, 34). Additionally, emerging evidence suggests that microbial dysbiosis in the gut-prostate axis may exacerbate systemic inflammation via regulatory T cell modulation and androgen receptor signaling, offering a potential mechanistic link warranting further investigation (35, 36).

Moreover, inflammatory biomarkers such as SIRI and PIV may interact with genetic alterations, such as PTEN loss, which are prevalent in PCa. For instance, PTEN loss promotes an inflammatory tumor microenvironment via PI3K/AKT signaling, potentially amplifying the effects of TAM-derived cytokines (e.g., IL-6) (16, 37). Integrating these biomarkers with genomic profiling could enhance risk stratification and guide targeted therapies. However, our study lacked genomic data, precluding direct analysis of these interactions and necessitating future research.

The U-shaped association of LMR with PCa risk, revealed by RCS analyses, suggests dual roles for inflammation. Low LMR, often indicative of lymphopenia or elevated monocyte counts, may reflect indeed reflect a state of immune suppression, where reduced lymphocyte-mediated anti-tumor surveillance allows for PCa initiation and progression (38, 39). Conversely, an excessively high LMR, driven by significant lymphocyte proliferation or a severe reduction in monocytes, could also represent a dysregulated immune response (40). While lymphocytes are typically anti-tumorigenic, an extreme proliferation might indicate chronic immune activation or an ongoing inflammatory process (e.g., certain chronic infections or autoimmune conditions) that paradoxically fuels tumorigenesis (41, 42). Such persistent immune imbalances can lead to the generation of genotoxic substances, contribute to chronic tissue repair and proliferation, or even trigger compensatory immune responses that ultimately create a pro-tumorigenic environment (41). Similarly, nonlinear relationships with tPSA and fPSA (e.g., minimum tPSA at LMR ~4.07) suggest that inflammatory states modulate PSA dynamics, potentially complicating PSA-based screening. These findings underscore the need for context-specific biomarker thresholds in PCa risk assessment.

ROC analyses demonstrated moderate discriminatory ability for PIV (AUC=0.709, 95% CI: 0.655–0.763) and SIRI (AUC=0.704, 95% CI: 0.650–0.759) in the clinical cohort, outperforming simpler indices like NLR and PLR. These composite biomarkers show promise for risk stratification, particularly for PCa, where PSA and multiparametric magnetic resonance imaging (mpMRI) have limitations, especially in the PSA “gray zone”. Integrating inflammatory biomarkers with PSA or mpMRI may enhance diagnostic accuracy and guide personalized immunotherapeutic strategies, such as targeting NF-κB or tumor-associated macrophages via CDK12/13 inhibition or CD47 blockade.

This study has limitations. The cross-sectional NHANES design limits causal inference, and reliance on PSA-based PCa definitions may introduce misclassification bias, as undiagnosed cases may be present in the non-PCa group, contributing to a milder disease spectrum. Furthermore, the exceptionally high AUCs for tPSA and fPSA in the NHANES cohort are closely tied to this cohort’s PCa definition, which may require validation with large-scale prospective data in the future. The stark PCa prevalence difference (7.0% in NHANES vs. 49.6% in the clinical cohort) reflects distinct populations, with the clinical cohort representing a pre-selected, high-risk group with more advanced disease, driving stronger associations and better predictive performance (spectrum effect). Inconsistent covariate adjustment—socioeconomic factors in NHANES versus clinical biochemical markers in the clinical cohort—may introduce residual confounding and limit direct comparisons. Ethnic differences between U.S. and Chinese populations may influence biomarker levels, and single-timepoint measurements may not capture dynamic inflammatory changes. Unmeasured confounders, such as infections or medications, could also affect results. Additionally, the clinical cohort did not fully collect all clinical variables, such as Gleason score, precluding in-depth exploration of associations between clinically significant PCa (Gleason Score ≥7) and inflammatory indices.

The strong associations of monocyte-inclusive indices (SIRI, PIV) with PCa risk and PSA levels suggest interplay with PCa’s endocrine drivers. Inflammatory cytokines (e.g., IL-6, TNF-α), often secreted by TAMs, modulate AR signaling, promoting AR overexpression in low-androgen environments and potentially driving castration-resistant PCa (39, 43). These findings suggest that targeting inflammatory pathways, particularly TAM-related mechanisms, could disrupt inflammation–endocrine crosstalk, offering novel therapeutic avenues.

Future research should pay more attention on prospective, multi-center studies to validate these biomarkers and establish clinical thresholds, accounting for ethnic variability and nonlinear associations. Integrating inflammatory biomarkers with genomic profiling or tumor microenvironment analyses could enhance risk stratification and personalization. To further elucidate the causal relationships and therapeutic potential of these pathways, interventional studies targeting inflammatory pathways are needed, which may include the administration of anti-inflammatory drugs, lifestyle modifications, or immunotherapies. Exploring the gut-prostate axis and its impact on systemic inflammation may further elucidate PCa pathogenesis and inform novel immunotherapeutic approaches.

## Conclusion

This study demonstrates consistent associations between systemic inflammatory biomarkers (SII, SIRI, PIV, NLR, PLR, LMR) and PCa risk, with nonlinear relationships to tPSA and fPSA, highlighting their potential as non-invasive tools for risk stratification. The superior performance of SIRI and PIV in the clinical cohort underscores the value of composite indices in capturing complex immune dynamics. These findings advance our understanding of inflammation-driven PCa and support the integration of inflammatory biomarkers into diagnostic and therapeutic strategies. Future research should focus on prospective validation, mechanistic elucidation, and targeted interventions to modulate inflammation for PCa prevention and management.

## Data Availability

The original contributions presented in the study are included in the article/**Supplementary Material**. Further inquiries can be directed to the corresponding authors.
