# Supplementary material for: Association of systemic inflammatory biomarkers with prostate cancer risk: a population-based (NHANES) and clinical validation study
Source: Front Endocrinol (Lausanne). 2025 Nov 10;16:1697617. doi: 10.3389/fendo.2025.1697617 (PMC12640860; doi:10.3389/fendo.2025.1697617)
Supplement: Supplementary file 1 [file Table1.docx]

**The results of missing data processing**

|  | **Before interpolation** | **After interpolation** | ***P.*overall** |
| --- | --- | --- | --- |
|  | ***N=7354*** | ***N=7354*** |  |
| Age | 59.0 [49.0;70.0] | 59.0 [49.0;70.0] | 1.000 |
| Race: |  |  | 1.000 |
| Mexican American | 1294 (17.6%) | 1294 (17.6%) |  |
| Other Hispanic | 441 (6.00%) | 441 (6.00%) |  |
| Non-Hispanic White | 4025 (54.7%) | 4025 (54.7%) |  |
| Non-Hispanic Black | 1334 (18.1%) | 1334 (18.1%) |  |
| Other Race | 260 (3.54%) | 260 (3.54%) |  |
| Education: |  |  | 1.000 |
| Below high school | 2307 (31.4%) | 2310 (31.4%) |  |
| High school or comparable | 1701 (23.2%) | 1702 (23.1%) |  |
| College or above | 3339 (45.4%) | 3342 (45.4%) |  |
| Marital: |  |  | 0.997 |
| Married/Living with partner | 5352 (72.9%) | 5359 (72.9%) |  |
| Widowed/Divorced/Separated/ | 1994 (27.1%) | 1995 (27.1%) |  |
| PIR | 2.55 [1.29;4.62] | 2.54 [1.29;4.62] | 0.911 |
| BMI | 28.0 [25.1;31.4] | 28.0 [25.1;31.4] | 0.708 |
| Alcohol: |  |  | 0.472 |
| No | 1234 (17.9%) | 1283 (17.4%) |  |
| Yes | 5652 (82.1%) | 6071 (82.6%) |  |
| Diabetes: |  |  | 1.000 |
| No | 6024 (82.0%) | 6029 (82.0%) |  |
| Borderline | 175 (2.38%) | 175 (2.38%) |  |
| Yes | 1150 (15.6%) | 1150 (15.6%) |  |
| SBP | 127 [116;139] | 127 [116;139] | 0.807 |
| DBP | 73.0 [66.0;81.0] | 73.0 [65.0;80.0] | 0.397 |
| NLR | 2.09 [1.56;2.81] | 2.09 [1.56;2.81] | 1.000 |
| SII | 487 [346;685] | 487 [346;685] | 1.000 |
| PLR | 122 [96.2;158] | 122 [96.2;158] | 1.000 |
| LMR | 3.50 [2.67;4.50] | 3.50 [2.67;4.50] | 1.000 |
| NPR | 0.02 [0.01;0.02] | 0.02 [0.01;0.02] | 1.000 |
| SIRI | 1.14 [0.78;1.67] | 1.14 [0.78;1.67] | 1.000 |
| PIV | 267 [174;409] | 267 [174;409] | 1.000 |
| Physical: |  |  | 1.000 |
| Inactive | 3535 (48.1%) | 3535 (48.1%) |  |
| Moderate | 2284 (31.1%) | 2284 (31.1%) |  |
| Vigorous | 615 (8.36%) | 615 (8.36%) |  |
| Both moderate and vigorous | 920 (12.5%) | 920 (12.5%) |  |
| tPSA | 1.00 [0.60;1.90] | 1.00 [0.60;1.90] | 1.000 |
| fPSA | 0.29 [0.18;0.49] | 0.29 [0.18;0.49] | 1.000 |
| ALT | 24.0 [19.0;32.0] | 24.0 [19.0;32.0] | 0.957 |
| AST | 25.0 [21.0;30.0] | 25.0 [21.0;30.0] | 0.981 |
| TG | 1.52 [1.02;2.34] | 1.52 [1.02;2.34] | 0.984 |
| Smoke: |  |  | 1.000 |
| Never | 2758 (37.5%) | 2760 (37.5%) |  |
| Former | 2899 (39.5%) | 2902 (39.5%) |  |
| Current | 1691 (23.0%) | 1692 (23.0%) |  |
| PCa: |  |  | 1.000 |
| No | 6840 (93.0%) | 6840 (93.0%) |  |
| Yes | 514 (6.99%) | 514 (6.99%) |  |

Abbreviations: PCa, Prostate cancer; PIR, Poverty income ratio; BMI, body mass index; WBC, white blood cell count; tPSA, Total prostatic specific antigen; fPSA, free prostatic specific antigen; ALT, Alanine aminotransferase; AST, Aspartate aminotransferase; TG, Triglycerides; SBP, Systolic blood pressure; DBP, Diastolic blood pressure.
